# Determination of the Infectious Agent of Translucent Post-Larva Disease (TPD) in *Penaeus vannamei*

**DOI:** 10.3390/pathogens9090741

**Published:** 2020-09-10

**Authors:** Ying Zou, Guosi Xie, Tianchang Jia, Tingting Xu, Chong Wang, Xiaoyuan Wan, Yingxia Li, Kun Luo, Xiaodong Bian, Xiuhua Wang, Jie Kong, Qingli Zhang

**Affiliations:** 1 Key Laboratory of Marine Aquaculture Disease Control, Ministry of Agriculture, Key Laboratory of Marine Aquaculture Epidemiology and Biosecurity, Yellow Sea Fisheries Research Institute, Chinese Academy of Fishery Sciences, Qingdao 266071, China; 15216481627@163.com (Y.Z.); xiegs@ysfri.ac.cn (G.X.); jiatianchang1@126.com (T.J.); xutingting83@163.com (T.X.); wangchongyilin@163.com (C.W.); wanxy@ysfri.ac.cn (X.W.); 18754809158@163.com (Y.L.); luokun@ysfri.ac.cn (K.L.); bianxd@ysfri.ac.cn (X.B.); wangxh@ysfri.ac.cn (X.W.); kongjie@ysfri.ac.cn (J.K.); 2 Laboratory for Marine Fisheries Science and Food Production Processes, Qingdao National Laboratory for Marine Science and Technology, Qingdao 266071, China

**Keywords:** infectious agent, *Vibrio parahaemolyticus*, translucent post-larvae disease (TPD), glass post-larvae disease (GPD), *Penaeus vannamei*

## Abstract

A new emerging disease called “translucent post-larvae disease” (TPD) or “glass post-larvae disease” (GPD) of *Penaeus vannamei*, characterized by pale or colorless hepatopancreas and digestive tract, has become an urgent threat to the shrimp farming industry. Following this clue that treatment of an antibacterial agent could alleviate the disease, systematic investigation of the potential infectious agent of TPD was conducted using bacterial identification and artificial challenge tests to fulfill Koch’s postulates. A dominant bacterial isolate, *Vp*-JS20200428004-2, from the moribund individuals was isolated and identified as *Vibrio parahaemolyticus* based on multi-locus sequence analysis. However, *Vp*-JS20200428004-2 differed from the *V. parahaemolyticus* that caused typical acute hepatopancreatic necrosis disease. Immersion challenge tests revealed that *Vp*-JS20200428004-2 could cause 100% mortality within 40 h at a dose of 1.83 × 10^6^ CFU/mL, and experimental infected shrimp showed similar clinical signs of TPD. The *Vp*-JS20200428004-2 could be re-isolated and identified from the experimental infected individuals. Moreover, histopathological analysis of diseased samples indicated that *Vp*-JS20200428004-2 caused severe necrosis and sloughing of epithelial cells of the hepatopancreas and midgut in shrimp individuals both naturally and experimentally infected. Our present results indicated that *Vp*-JS20200428004-2 is a highly virulent infectious agent associated with the TPD and deserves further attention.

## 1. Introduction

Aquaculture, including shrimp farming, is the fastest-growing sector producing food of animal origin in the world [1]. Several emerging diseases, including acute hepatopancreatic necrosis disease (AHPND), viral covert mortality disease (VCMD), infection by Enterocytozoon hepatopenaei (EHP), and infection of shrimp hemocyte iridescent virus (SHIV), have impacted on the global shrimp aquaculture industry [1,2,3,4,5]. More recently, a novel disease called “translucent post-larvae disease” (TPD) or “glass post-larvae disease” (GPD) in *Penaeus vannamei* has become a rapidly growing threat to shrimp farming in China recently [6,7].

Since March 2020, an extensive number of cases of TPD has occurred in some hatcheries of *P. vannamei* in Guangdong and Guangxi provinces, after which this new disease began to spread to major shrimp farming areas in the north of China via post-larvae (PL) transportation in April 2020 [6,7]. Translucent post-larvae disease mostly affects post-larvae at four to seven days old (PL4~PL7) with very severe infectivity. Usually, the morbidity of a diseased population can reach up to 60% on the second day after first observing abnormal individuals, and even up to 90–100% in severe cases on the third day. Translucent post-larvae disease affected shrimp mainly show similar gross clinical signs, such as a pale or colorless hepatopancreas and empty digestive tract, which causes the body of diseased individuals to become transparent and translucent; therefore, these diseased individuals were named “translucent post-larvae” or “glass post-larvae” by local farmers.

Since TPD disease has become prevalent in farmed shrimp stocks, leading to serious economic losses in some shrimp farming areas in China, it is an urgent necessity to investigate and develop strategies for preventing the disease. We sampled TPD shrimp and screened for the known pathogens, but PCR assays indicated that TPD shrimps were free of known shrimp viral pathogens, including white spot syndrome virus (WSSV), infectious hypodermal and hematopoietic necrosis virus (IHHNV), *V*_AHPND_, SHIV, yellow head virus (YHV), Taura syndrome virus (TSV), and infectious myonecrosis virus (IMNV). These results suggested that TPD might be caused by a new emerging pathogen. In addition, some farmers found that treatment of water in rearing tanks with an antibacterial agent could alleviate the disease. This clue suggested that the disease might be caused by a bacterial pathogen. Therefore, a systematic investigation of the potential infectious agent causing TPD was conducted based on bacterial isolation and identification, challenge experiments, and histopathological analysis, the results of which are presented in this study.

## 2. Results

### 2.1. Clinical Signs

**Figure 1:**
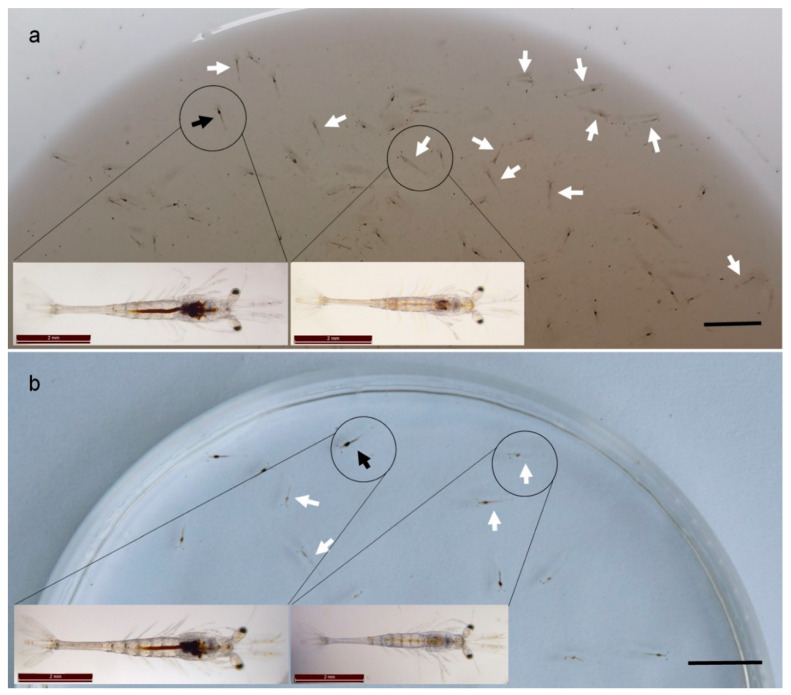
Clinical signs of the *Penaeus vannamei* affected by the translucent post-larvae disease (TPD). (**a**) The *P. vannamei* individuals collected from TPD-affected rearing tanks of post-larvae. (**b**) The *P. vannamei* individuals from the immersion challenge bioassay. All the samples were at PL7 stage, and body length was approximately 0.6–0.9 cm. The diseased individuals (indicated by the white arrows) demonstrated syndromes of abnormal hepatopancreas and digestive tract necrosis. The hepatopancreas and digestive tract of the diseased post-larvae were pale and colorless. The bar scales are 10 mm and 2 mm in (**a**,**b**) and the magnified images, respectively.

The clinical signs of diseased shrimps, either from experimental challenge tests or naturally affected rearing tanks of TPD, were similar, with abnormal hepatopancreases and digestive tracts as shown in Figure 1. The hepatopancreases and digestive tracts of the diseased post-larvae shrimp became pale and colorless (Figure 1a). A high percentage of individuals of the affected post-larvae sunk to the bottom of the rearing tanks because of the decreased swimming capability caused by the disease.

### 2.2. Detection of Known Pathogens in the Diseased Samples

The detection results showed that the eight known shrimp pathogens, including *V*_AHPND_, WSSV, IHHNV, *V*_AHPND_, EHP, SHIV, YHV, TSV, and IMNV, were all negative in the shrimp samples suffering TPD (Supplementary Figure S1). These results suggested that the TPD-shrimp might be affected by a new emerging pathogen.

### 2.3. Bacterial Identification

**Figure 2:**
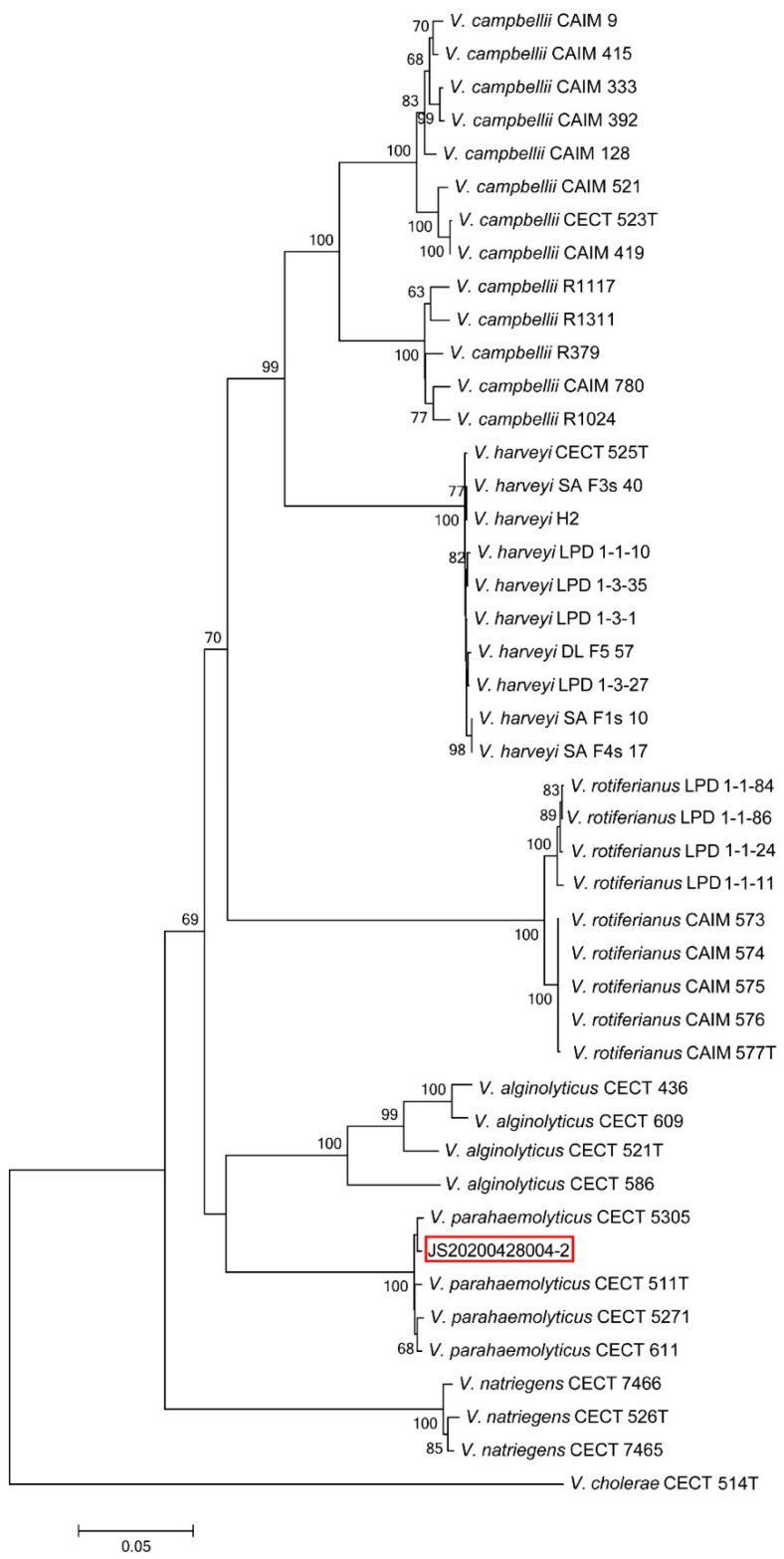
Phylogenetic reconstruction based on concatenated *rpoD*, *rctB*, and *toxR* sequences of the JS20200428004-2 strain. Percentage bootstrap values (1000 replicates). Bar = 0.05. The reference sequences were as described by Pascual et al. [8].

**Figure 3:**
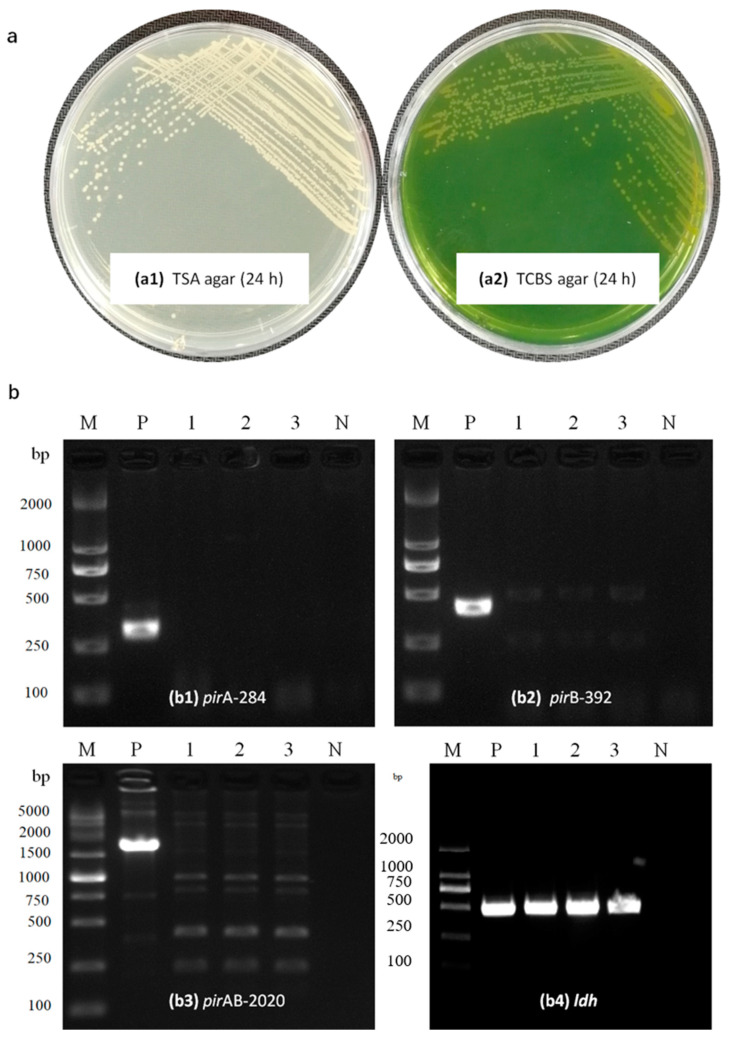
Bacterial identification and analysis of virulence genes. (**a**) The color of *Vp-*JS20200428004-2 cultured on trypticase soy (TSA) and Thiosulfate Citrate bile salts sucrose (TCBS) agar. (**b**) Electrophoretogram of molecular detection of toxin gene *pir*A (b1), *pir*B (b2), *pir*AB2020*^VP^* (b3), and *ldh* (b4) of collected strain in this study. *p*: positive control; N: negative control; 1-3: JS20200428004-2. M: Molecular marker.

The sequences of the PCR products were subjected to BLAST. Analysis of its 16S rRNA gene sequence showed that all of the 10 dominant strains including JS20200428004-2 belonged to the genus *Vibrios* with the highest similarity to *V. parahaemolyticus* (99.93%). Further determination of bacterial taxonomy was carried out by multi-locus sequence analysis (MLSA). The multi-locus sequence concatenated sequences of the *rpoD*–*rctB*–*toxR* genes analysis also clearly identified strain JS20200428004-2 as being the closest to *V. parahaemolyticus* (Figure 2). The colony of JS20200428004-2 showed the color of milk white on the TSA agar with a neat edge and raised surface, and the diameter was 1.79 mm after incubation for 24 h at 28 °C; however, the colony was faint yellow on TCBS agar, and the diameter was 2.04 mm after incubation for 24 h at 28 °C (Figure 3a). The result of bacteria identification revealed that the post-larvae *P. vannamei* might be the carrier or infected by *Vp*-JS20200428004-2. Meanwhile, the dominant colony isolated from the moribund post-larvae in the challenge test was determined to be completely consistent with the *Vp*-JS20200428004-2 based on analysis of the 16S rRNA and *toxR* gene, which confirms that the bacteria functioning in the challenge test was the strain of *Vp*-JS20200428004-2. The result of the biochemical test showed that the parameters of *Vp*-JS20200428004-2 were identical to those of *Vibrio* (Supplementary Table S1).

### 2.4. Virulence Genes in the Isolated Strain

The 450 bp PCR amplicon of the *ldh* gene from *Vp*-JS20200428004-2 (Figure 3(b4)) was sequenced and submitted to the NCBI website for BLAST analysis. The results showed that the strain *Vp*-JS20200428004-2 carried the *ldh* gene (also named the thermolabile hemolysin gene, *tlh*) and shared a 100% homology with the known *tlh* genes of *V. parahaemolyticus*. Subsequently, the primer sets of *pir*A-284F/R and *pir*B-392F/R produced the targets’ amplicons by using the DNA of the standard *V*_AHPND_ isolate as a template, whereas failed to amplify the DNA of the *Vp*-JS20200428004-2 strain (Figure 3(b1,b2)). In addition, the entire PirAB*^Vp^* toxin gene tandem plus a portion of their upstream and downstream regions of 2020 bp were obtained from standard *V*_AHPND_ isolate using the primer *pir*AB2020-F/R; however, the same primers failed to produce the PCR amplicon when using the DNA of *Vp*-JS20200428004-2 as a template (Figure 3(b3)). The results of the PirAB*^Vp^* toxin gene assays indicated that the isolated strain of *Vp*-JS20200428004-2 lacked the *pir*A*^Vp^* and *pir*B*^Vp^* genes.

### 2.5. Pathogenicity of the Vibrio. Parahaemolyticus Determined by the Challenge Test

**Figure 4:**
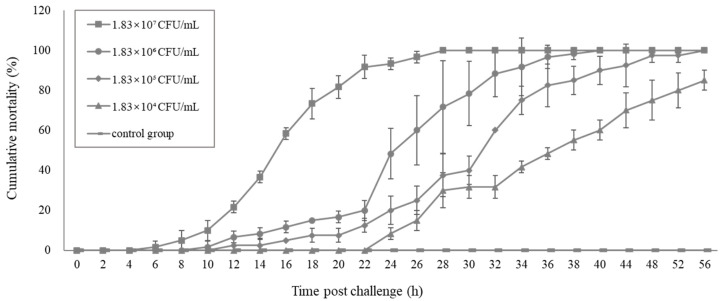
Cumulative mortality of *Penaeus vannamei* induced by immersion in gradient concentration of bacteria *Vp*-JS20200428004-2. Four groups of healthy shrimp were immersed in a dilution concentration of 1.83 × 10^7^ ~ 1.83 × 10^4^ CFU/mL (infected group), and one group was immersed in boiled seawater (control group). The cumulative mortalities of shrimp are shown as the means and SD of data from two replicates for each experimental group.

The mortality rate of post-larvae *P. vannamei* infected with a concentration of 1.83 × 10^7^ CFU/mL was obviously the highest among all the infected groups, and 100% mortality was observed in the 28 h post challenge. One hundred percent mortality in the group of 1.83 × 10^6^ CFU/mL occurred at 40 h post challenge. Finally, 75% mortality in the group of 1.83 × 10^4^ CFU/mL, the lowest pathogen concentration used in the challenge tests in this study, occurred at 48 h post challenge. In contrast, there was no mortality in the negative control group during the experiment period (Figure 4). In the challenge test, the lethal concentration of 50% (LC50) at 24 h was 9.84 × 10^5^ CFU/mL, and the lethal times of 50% (LT_50_) in these 56 h challenge tests with a dose of 1.83 × 10^7^, 1.83 × 10^6^, 1.83 × 10^5^, and 1.83 × 10^4^ CFU/mL were 15.7 h, 24.6 h, 28.9 h, and 38.6 h, respectively.

Meanwhile, most of the individual shrimp in the challenged group showed the typical clinical signs of TPD, including pale, colorless hepatopancreas and midgut. Meanwhile the diseased individuals showed obvious signs of being lack of food in the hepatopancreas and digestive tract at high magnification (Figure 1b).

### 2.6. Histopathology Analysis of the Vibrio Infection

**Figure 5:**
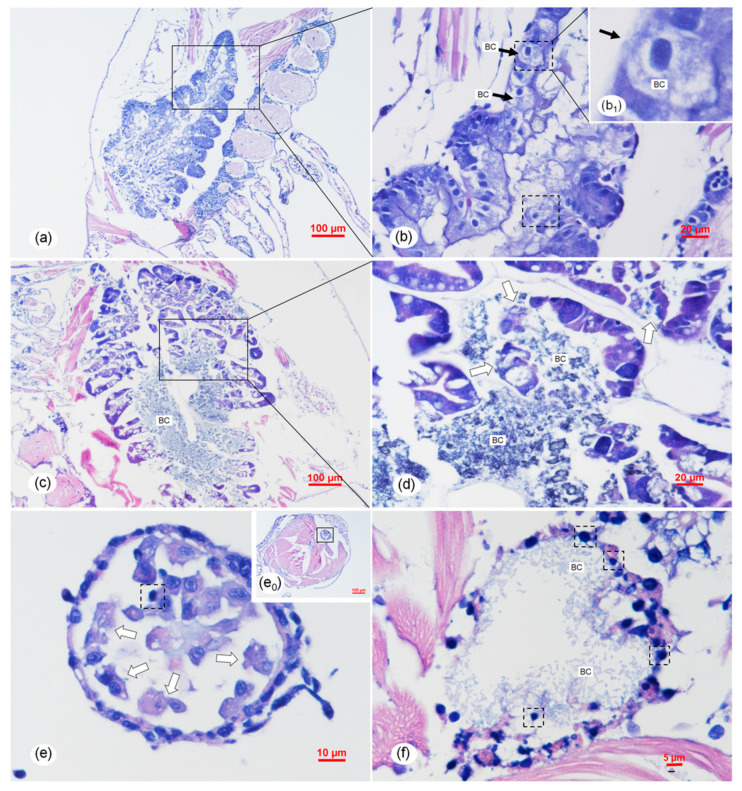
Histological sections of the naturally infected post-larvae in shrimp rearing tanks of post-larvae suffering from TPD. (**a**,**b**) Early phase with active destruction of the hepatopancreas. Note the mild necrosis of epithelial cells (ECs) of hepatopancreatic (HP) tubules, especially the ECs in the dotted boxes showing typical dark, smaller, and condensed nuclei. The bacterial colonization (BC) in the early phase of infection was indicted by the black arrows. The arrowed ECs (black arrow) were B-cells with the large vacuoles. (**c**,**d**) Acute phase with massive bacterial invasion. There was vast bacterial invasion of the hepatopancreatic tubules in half of the organs, where the bacterial masses and the tubules were destroyed. Note the detachment/sloughing of hepatopancreatic ECs (white arrows). (**e**,**f**) Midgut of an affected digestive tract of a naturally infected post-larvae showing necrosis (dotted box) and sloughing (white arrows) of ECs of the digestive tract. Note the mass BC in the tubule lumens of the digest tract at the midgut. (**b**), (b1), (**d**), and (**e**) are the magnified micrographs of the area in the black frames in (**a**–**c**), and (e_0_), respectively. (**a**,**b**,**e**) show the pathological change in the early phase of infection. (**c**,**d**,**f**) show the pathological change in the acute phase of infection. Scale bars = (**a**) 100 μm, (**b**) 20 μm, (**c**) 100 μm, (**d**) 20 μm, (e_0_) 100 μm, (**e**) 10 μm, and (**f**) 5 μm.

**Figure 6:**
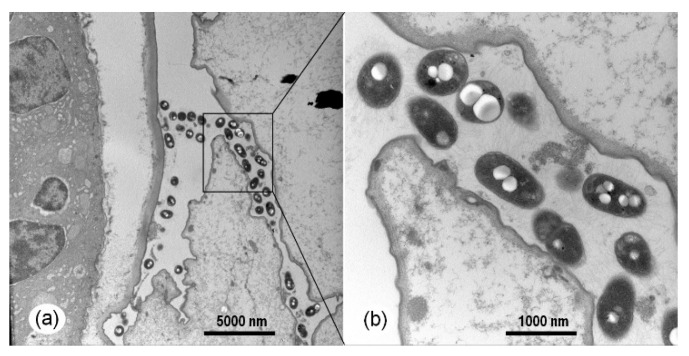
The bacterial colonization in the HP of the naturally infected post-larvae in shrimp rearing tanks of post-larvae suffering from TPD under transmission electron microscopy. (**b**) The magnified micrographs of the area in the black frames in (**a**).

Histopathological examination of the naturally infected individuals showed that necrosis, as well as sloughing of the epithelial cells, occurred in the hepatopancreatic tubules and midgut (Figure 5). In the early phase of infection, necrosis of epithelial cells was minor in the hepatopancreatic tubules (Figure 5a,b), but necrosis and sloughing of epithelial cells were severe in the midgut (Figure 5e). Necrosis and sloughing of epithelial cells became very severe, and typical signs of significant bacterial colonization both in the hepatopancreas tubule lumens and the midgut lumens could be observed in the acute and late phase of infection (Figure 5c,d,f). The bacterial colonization in the HP was verified by using the transmission electron microscopy analysis (Figure 6a,b). All of the observations were almost identical in the hepatopancreases and digest tracts of sick individuals sampled from different regions. The H&E figures of the normal shrimp from rearing tanks affected by TPD is supplied in Supplementary Materials Figure S2 as a comparison.

**Figure 7:**
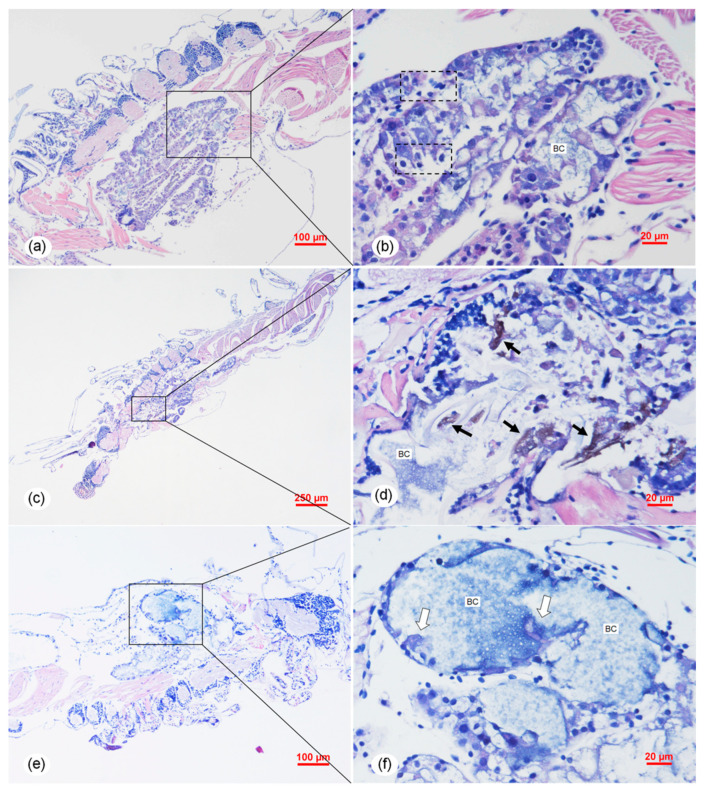
Histological sections of the artificially infected post-larvae in immersion bioassay showing similar TPD lesions in hepatopancreas (HP) induced by *Vp*-JS20200428004-2 isolate. (**a**,**b**) Early phase with active destruction of the HP. Affected HP tubules showed obvious necrosis of epithelial cells (ECs) (dotted boxes) with typical dark, smaller, and condensed nuclei. (**c**,**d**) Middle phase with obvious melanization (black arrows) and bacterial colonization (BC) in the HP. (**e**,**f**) Acute (late) phase with massive bacterial invasion. Huge numbers of bacteria occupied the hepatopancreatic tubules. Note the detachment/sloughing of hepatopancreatic ECs (white arrows). (**b**,**d**,**f**) are the magnified micrographs of the area in the black frames in (**a**,**c**,**e**), respectively. Sloughing of the ECs in the HP tubule are indicated by the white arrows. Note the BC in epithelial cells in the HP tubule lumens. Scale bars = (**a**) 100 μm, (**b**) 20 μm, (**c**) 250 μm, (**d**) 20 μm, (**e**) 100 μm, and (**f**) 20 μm.

**Figure 8:**
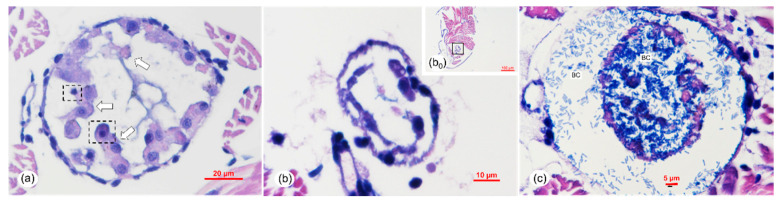
Histological sections of a digestive tract from an artificially infected post-larvae in an immersion bioassay. (**a**) Early phase of the affected digestive tract at the midgut of the artificially infected post-larvae showing necrosis (dotted boxes) and sloughing of epithelial cells (ECs) of the digestive tract (white arrows). (**b**) Middle phase with severe necrosis of ECs of the digestive tract. (**c**) Late phase with BC. (**b**) was a magnified micrograph of the area in the black frames in (b_0_). Scale bars = (**a**) 20 μm, (b_0_) 100 μm, (**b**) 10 μm, and (**c**) 5 μm.

Histopathology of the individuals in the immersion challenge test of *V. parahaemolyticus* (*Vp*-JS20200428004-2) indicated that HP tubule epithelial cells showed necrosis and sloughing into the HP tubule lumens (Figure 7a–d). In the early phase, the necrosis and sloughing of epithelial cells also appeared in the digestive tract at the midgut (Figure 8a). As the bacterial infection became more serious, severe necrosis and sloughing of epithelial cells were observed in both the hepatopancreatic tubules (Figure 7c,d) and the digestive tract at the midgut (Figure 8b) in the middle phase of infection. The melanization caused by the bacterial infection was obvious in the hepatopancreatic epithelial cells (Figure 7d), and the same symptom of melanization also appeared in the hepatopancreatic epithelial cells of the naturally infected shrimps (Supplementary Figure S3). Obvious signs of bacterial colonization also occurred in both of the HP tubule lumens (Figure 7f) and the digest tract (Figure 8c) in the late phase of infection.

## 3. Discussion

Over the past decade, the world shrimp farming industry has been heavily affected by several emerging and reemerging diseases [1,9,10]. One of those major diseases is AHPND, also known as early mortality syndrome (EMS) [9,11,12]. AHPND has spread to other shrimp farming countries since it was first reported in Vietnam and China in 2010. The global prevalence of AHPND has caused significant losses in shrimp production in Thailand, Vietnam, China, Malaysia, and Mexico [13,14,15,16], and the direct global economic loss caused by AHPND was over US$ 44 billion from 2010 to 2016 [17,18,19].

The causative agent of AHPND proved to be some unique isolates of *Vibrio* (*V*_AHPND_) which carry a plasmid of approximately 70 kb carrying genes for toxins that resemble the binary Photorhabdus insect-related (Pir) toxins PirA and PirB [13,20,21,22]. The pathognomonic lesions of AHPND were characterized by massive sloughing of tubule epithelial cells of the shrimp hepatopancreas (HP) [23,24,25,26]. At the very beginning, *V*_AHPND_ was deduced to be the causative agent of TPD because the gross clinical signs of the diseased post-larvae were similar to AHPND’s syndrome to some degree. However, both the AP4 nested PCR method and duplex *pir*AB*^Vp^* PCR assays of the TPD-shrimp samples showed negative results for the *V*_AHPND_ test. In addition, AHPND occurs approximately within 35 days after stocking of shrimp ponds, and the onset of clinical signs and mortality of AHPND start as early as 10 days post-stocking in some extreme cases [27] (. Whereas, TPD usually occurs in post-larvae of 4 to 7 days, which is much earlier than when AHPND usually appears. Hence, we deduced that TPD was a novel disease that differed from AHPND.

The results of molecular assays showed that the diseased individuals in the TPD-affected rearing tanks of post-larvae were negative for WSSV, IHHNV, *V*_AHPND_, EHP, SHIV, YHV, TSV, and IMNV. In consideration that treatment of water in rearing tanks with an antibacterial agent could alleviate the disease, we conducted bacterial pathogen isolation and identification from the diseased *P. vannamei* samples suffering from TPD first. The dominant bacteria isolate from the moribund infected shrimp were identified as *V. parahaemolyticus* according to sequence alignment and evolutionary tree analysis based on the 16S rDNA and three toxin gene sequences. Pathogenicity analysis of the *V. parahaemolyticus* in the challenge test indicated that the typical gross clinical signs were similar to those of farm-based TPD. The onset and progression of the disease in the challenge test as well as the mortality were also similar to those of farmed-based TPD. Thus, the novel *V. parahaemolyticus* (*Vp*-JS20200428004-2) was deduced to be the pathogenic agent of TPD in post-larvae of shrimp.

Two types of deletion mutants regarding the *pir*AB*^Vp^* genes in *V. parahaemolyticus* have been found from AHPND-affected farms [28]. The type I mutants include three strains with deletion of entire *pir*AB*^Vp^* genes. The type II mutants include three strains with smaller deletion of the *pir*A*^Vp^* gene and partial *pir*B*^Vp^* gene. These two types of *V. parahaemolyticus* were not pathogenic to shrimp which indicated both *pir*A*^Vp^* and *pir*B*^Vp^* were related to AHPND pathogenicity. Recently, a *V. parahaemolyticus* isolate (XN87) lacking *pir*A*^Vp^* but carrying *pir*B*^Vp^* was identified, and it could cause 47% mortality without AHPND lesions in shrimp immersion challenge [27]. Sequence and virulence analysis revealed that XN87 carrying mutant pVA plasmids that produce no Pir*^Vp^* toxins could cause mortality in shrimp in ponds. In 2020, Andrea et al. [29] reported that there is a proportion of *Vibrio* isolates (isolates 36 and 43) harboring intact *pir*AB*^Vp^*, but they did not produce AHPND PirAB*^Vp^* toxins. Atypical *V. parahaemolyticus* isolates (isolates 36 and 43) produced neither PirAB*^Vp^* toxins nor AHPND pathology but still killed shrimp. All of the abovementioned reports revealed that the pathogenic mechanism of typical *V*_AHPND_ and atypical *V. parahaemolyticus* is more complicated than the initial understanding of researchers. The pVA plasmid does not always determine the pathogenicity of *V. parahaemolyticus* to shrimp, and the presence of pirAB*^Vp^* toxin genes is insufficient for confirmation of AHPND-causing bacteria. In the present study, results of virulent genes analysis showed that the isolate of *Vp*-JS20200428004-2 lacks of the *pir*A*^Vp^* and *pir*B*^Vp^* genes; however, it possesses the *ldh* gene which encodes a key virulent factor, thermolabile hemolysin, and is lethal to shrimp. In addition, *Vp*-JS20200428004-2 of 1.83 × 10^6^ CFU/mL caused 100% mortality within 40 h post challenge; that is, the isolate of *Vp*-JS20200428004-2 seemed to be more virulent than both the typical *V*_AHPND_ and atypical *V. parahaemolyticus* previously reported. These results revealed the higher diversity of pathogenic *V. parahaemolyticus* in shrimp.

Acute necrosis of HP epithelial cells and sloughing of the HP epithelial cells occurred in the hepatopancreatic tubules from both naturally and the artificially infected post-larvae individuals. The gross histopathological signs of TPD was not identical to those of AHPND, of which there was massive cell sloughing of hepatopancreatic tubule epithelial cells [13,28]. Meanwhile, the histopathological signs of TPD were also different from those caused by atypical *V. parahaemolyticus* [29,30]. The unique gross histopathological signs of TPD suggested that the pathogenic mechanism of *Vp*-JS20200428004-2 was different from those of both typical *V*_AHPND_ and atypical *V. parahaemolyticus*. For further exploration of the pathogenic mechanism of *Vp*-JS20200428004-2, more systematic comparative studies on the *Vp*-JS20200428004-2, typical *V*_AHPND_, and atypical *V. parahaemolyticus* should be designed and conducted; details about the functional genes, plasmids, and genome of *Vp*-JS20200428004-2 should especially be further investigated in the future.

So far, vibriosis, caused by various *V. spp,* has been reported to occur at different developmental stages of *P. vannamei* including the naupllii (2 days), zoea (4–5 days), mysis (3–5 day), post-larvae (10–15 days), and juveniles [31,32]. *Vibro alginolyticus* was associated with the zoea 2 syndrome and the mysis mold syndrome in the stage of larvae, while *V. alginolyticus* and *V. harveyi* were associated with the bolitas syndrome [32]. The bolitas syndrome was referred to a larvae syndrome in *P. vannamei* involving the detachment of epithelial cells from the intestine and hepatopancreas, which appeared as small spheres within the digestive tract [31]. Apparently, the histopathological syndromes of the intestine and hepatopancreas of TPD caused by the *Vp*-JS20200428004-2 were different from that of bolitas syndrome. In addition, the clinical signs of TPD were distinct from the clinical signs of bolitas syndrome, which included reduced feeding, retarded development, sluggish swimming, and reduced escape mechanism [31]. Thus, TPD was deduced to be a novel vibrio disease occurred in the stage of post-larvae of *P. vannamei*.

In summary, based on the systematic analysis including pathogenic agent isolation, identification and assays following the four criteria of Koch’s postulates, we confirmed that a novel *V. parahaemolyticus* (*Vp*-JS20200428004-2) was the causative agent associated with the emerging TPD that affected shrimp farming in 2020. The new pathogen shows high virulence to the post-larvae of shrimp and can cause acute and severe histopathological changes in the hepatopancreas and midgut. The risk of epidemic disease and losses in post-larvae caused by this new pathogen deserves further attention.

## 4. Materials and Methods

### 4.1. Sample Collection and Experiment Shrimp

The diseased post-larvae *P. vannamei* (PL7, body length 6–8 mm) were sampled from a shrimp farm in Ganyu, Jiangsu Province, on 28 April 2020. The moribund individuals were fixed in 4% paraformaldehyde for histopathological examination and 2.5% glutaraldehyde solution for transmission electron microscope observation, respectively. The healthy post-larvae shrimp (PL3, body length 4–6 mm) were purchased from a shrimp farm in Weifang, Shandong Province, and reared for two days then used for the challenge test.

### 4.2. Detection of Eight Known Shrimp Pathogens in the Diseased Samples

The presence of 8 known shrimp pathogens (including WSSV, IHHNV, *V*_AHPND_ (AP4-F/R primers), EHP, SHIV, YHV, TSV, and IMNV) in the diseased samples was detected by using the recommended methods in the Manual of Diagnostic Tests for Aquatic Animals [33]. In addition, the detection of SHIV and EHP was conducted according to the previously reported protocols [5,34]. The total DNA was extracted from the samples preserved in 95% ethanol by TIANamp Marine Animals DNA Kit (Tiangen, Beijing, China) according to the manufacturer’s protocol. Total RNA was extracted by using the QIAamp viral RNA kit (Qiagen Sciences, Gaithersburg, Maryland). The concentration and quality of extracted DNA/RNA was assessed by NanoDrop 2000c Spectrophotometer at wavelengths of 260 and 280 nm (Thermo Scientific, Waltham, MA, USA).

### 4.3. Bacteria Isolation

After disinfection with 75% alcohol and followed with three washes of PBS buffer (pH 7.2; Solarbio), the moribund shrimp from rearing tanks were homogenized in 0.5 mL of sterile PBS buffer. Marine 2216 agar was used for bacterial isolation and purification at 28 °C. Trypticase soy agar supplemented with 2% NaCl (TSA^+^) was used for the subculture of bacteria at 28 °C. The isolated bacteria were stored in Marine 2216 broth containing 15% (*v/v*) glycerol at −80 °C. The isolation protocol was also applied for re-isolation of dominant strains from the shrimp in the challenge test.

### 4.4. Bacteria Identification

A distinct single colony was selected from the TSA^+^ plate and re-suspended in 50 µL sterile water, then boiled at 95 °C for 10 min. After centrifugation at 3000*× g* for 30 s, the supernatant was used as template for PCR detection.

For bacteria identification, we applied 16S rRNA gene sequence analysis which provided a genus-level taxonomy of the strain firstly, and then MLSA based on three protein-coding genes (*rct*B, *rpo*D, and *tox*R) was applied for identification of strain according to the method described by Pascual et al. [8]. The concatenated sequences of *rpo*D–*rct*B–*tox*R sequences alignment was processed, and the resultant tree topology was evaluated using bootstrap analysis based on the neighbor-joining algorithm with 1000 replicates which was embedded in MEGA7.

The universal primers for detecting 16S rDNA of the bacteria used in this study were 27F and 1492R (Table 1) according to previous studies [35,36]. The PCR reaction mixture (25 µL) for 16S rDNA consisted of 12.5 µL ExTaq premix (Takara, Dalian, China), 1 µL of each primer (10 mM), and 1 µL DNA template. The amplicons of PCR were obtained from the following reaction: 95 °C for 5 min, followed by 30 cycles of 95 °C for 30 s, 55 °C for 30 s and 72 °C for 1 min 20 s with a final extension step at 72 °C for 7 min.

According to previous reports [8], the sequences of conserved *rct*B and *rpo*D genes were obtained by a thermal program: (i) 95 °C for 5 min; (ii) 10 cycles of 95 °C for 1 min; 54 °C for 2 min; 72 °C for 1 min 15 s; (iii) 25 cycles of 95 °C for 35 s; 54 °C for 2 min; 72 °C for 1 min15 s; (iv) extension step at 72 °C for 10 min, and the amplification of *tox*R fragment was completed by the touchdown PCR method: (i) 95 °C for 5 min; (ii) 8 cycles of 95 °C for 1 min; 62 °C for 2 min; 72 °C for 1 min 15 s, with 1 °C drop per cycle; (iii) 27 cycles of 95 °C for 35 s; 54 °C for 2 min; 72 °C for 1 min 15 s; (iv) extension step at 72 °C for 7 min.

The biochemical tests of *Vp*-JS20200428004-2 were conducted using API 20NE (BIOMERIEUX, Marcy I’Etoile, France) after incubating for 48 h according to the protocol in previous reports [38,39].

### 4.5. Analysis of Virulent Genes in the Isolated Strain

The virulent genes, lecithin-dependent haemolysin gene (*ldh*) and *pir*AB2020*^Vp^*, in *V. parahaemolyticus* were detected according to previous report [29]. The target genes *pir*A*^Vp^* (284 bp) and *pir*B*^Vp^* (392 bp) were amplified according to Han et al. [24]. All the involved primers are listed in Table 1, and the *ldh*-specific PCR was carried out at 94 °C for 5 min followed by 35 cycles at 94 °C for 30 s, 52 °C for 30 s, and 72 °C for 30 s and a final cycle at 72 °C for 7 min. The target gene *pir*AB2020*^Vp^* was amplified by 35 cycles at 94 °C for 40 s, 50 °C for 40 s, and 72 °C for 2 min 10 s after denatured for 5 min at 95 °C, and a final cycle at 72 °C for 7 min according to the OIE manuals [33].

### 4.6. Experimental Challenge by Immersion

The healthy post-larvae *P. vannamei* were cultivated temporarily in sterile seawater (21 °C) for two days and then randomly divided into 5 groups (i.e., four infected groups and one control group), 20 tails/group with two replications for each group. The average body length of the shrimp was 5.5 mm ± 0.2 mm (*n* = 10). The immersion challenge test was carried out as previously described by Tran et al. [13] with modifications.

To prepare the bacterial inoculum used in the challenge test, the *Vibrio* strain JS20200428004-2 was incubated in Tryptic Soy Broth medium with extra NaCl (TSB^+^) overnight at 28 °C. The concentration of bacterial solution was adjusted to the value 1.0 of OD_600_ (approximately corresponding to 10^9^ cells/mL) in a microplate reader (Don Whitley Scientific Limited, Shipley, West Yorkshire, England) and then confirmed by the plate colony counting method.

The obtained bacteria were centrifuged at 6000 rpm for 10 min at 4 °C, washed and re-suspended three times using sterile seawater, and finally diluted to 1.83 × 10^7^–1.83 × 10^4^ CFU/mL in boiled seawater as the infected group. The shrimp immersed in 1000 mL boiled seawater were only used as the control group. Mortalities were monitored every two hours for 24 h and median lethal concentrations (LC_50_) were then calculated. The dead and moribund shrimps were removed, and the moribund shrimps were fixed in 4% paraformaldehyde, and then bacteria were re-isolated and verified following the same protocol as the method described above.

### 4.7. Histopathology

The fixed shrimp with 4% PFA–PBS fixative from rearing tanks and the infected group in the laboratory were embedded with paraffin immediately after being dehydrated with a gradient of ethanol solutions. One slide of each sample was stained with H&E according to the routine histological procedures described by Lightner (1996) [40]. Analysis of transmission electron microscopy was conducted according to the protocol in a previous report [4].

## Figures and Tables

**Table 1:** Primers sequences of *rct*B, *rpo*D, *tox*R, *ldh*, and *pir*AB*^Vp^* for PCR.

Gene	Primer	Primer Sequences (5′-3′)	References
16S rDNA	27F	AGAGTTTGATCMTGGCTCAG	[35,36]
	1492R	GGYTACCTTGTTACGACTT	
*rpo*D	*rpo*D-F	ACGACTGACCCGGTACGCATGTAYATGMGNGARATGGGNACNGT	[8]
	*rpo*D-R	ATAGAAATAACCAGACGTAAGTTNGCYTCNACCATYTCYTTYT	
*rct*B	*rct*B -F	ATHGARTTYACNGAYTTYCARYTNCAY	
	*rct*B-R	YTTNCTYTGHATNGGYTCRAAYTCNCCRTC	
*tox*R	*tox*R-F	GANCARGGNTTYGARGTNGAYGAYTC	
	*tox*R-R	TTDKKTTGNCCNCYNGTVGCDATNAC	
*ldh*	*ldh*-F	AAAGCGGATTATGCAGAAGCACTG	[29]
	*ldh*-R	GCTACTTTCTAGCATTTTCTCTGC	
*pir*A*^Vp^*	*pir*A-284F	TGACTATTCTCACGATTGGACTG	[21]
	*pir*A-284R	CACGACTAGCGCCATTGTTA	
*pir*B*^Vp^*	*pir*B-392F	TGATGAAGTGATGGGTGCTC	
	*pir*B-392R	TGTAAGCGCCGTTTAACTCA	
*pir*AB*^Vp^*	*pir*AB2020-F	GCACCGTAAATTTTCAGGTT	[27]
	*pir*AB2020-R	CGTTGCAATCTAAGACATAG	
AP4	AP4-1	AP4-F1: ATGAGTAACAATATAAAACATGAAAC	[37]
		AP4-R1: ACGATTTCGACGTTCCCCAA	
	AP4-2	AP4-F2: TTGAGAATACGGGACGTGGG	
		AP4-R2: GTTAGTCATGTGAGCACCTTC	

## Supplementary Materials

The following are available online at https://www.mdpi.com/2076-0817/9/9/741/s1, Figure S1. Electrophoretogram of molecular detection of eight common pathogens in the diseased individuals of the translucent post-larvae disease (TPD) case. M: molecular marker; P: positive control; N: negative control; S: sample. (f1) showed the first step PCR amplicon of nested PCR for each pathogen. (f2) showed the second step PCR amplicon of the nested PCR for each pathogen. AP4 primer set was used in the AHPND test. M: Molecular markers, Figure S2. Histological sections of the normal shrimp from rearing tanks affected by TPD. (a) micrograph of low magnification. (b) The magnified micrographs of the area in the black frames in (a). Note the intact hepatopancreatic tubules (HTs) and epithelial cells of HTs. Scale bars = (a) 100 μm, (b) 20 μm, Figure S3. Histological sections of the hepatopancreatic (HP) tubules from the shrimp natural infected with TPD. (a, b) The hepatopancreas tubules infected by bacteria showed melanization (black arrows). (c, d) The massive bacterial colonization (BC) in epithelial cells and tubule lumens of the HP. Scale bars = (a) 100 μm, (b) 20 μm, (c) 100 μm, (d) 20 μm, Table S1. The results of biochemical test for *Vp*-JS20200428004-2 by API 20NE.
