# Supplementary material for: Determination of the Infectious Agent of Translucent Post-Larva Disease (TPD) in Penaeus vannamei
Source: Pathogens. 2020 Sep 10;9(9):741. doi: 10.3390/pathogens9090741 (PMC7558154; doi:10.3390/pathogens9090741)
Supplement: Supplementary file 1 [file pathogens-09-00741-s001.pdf]

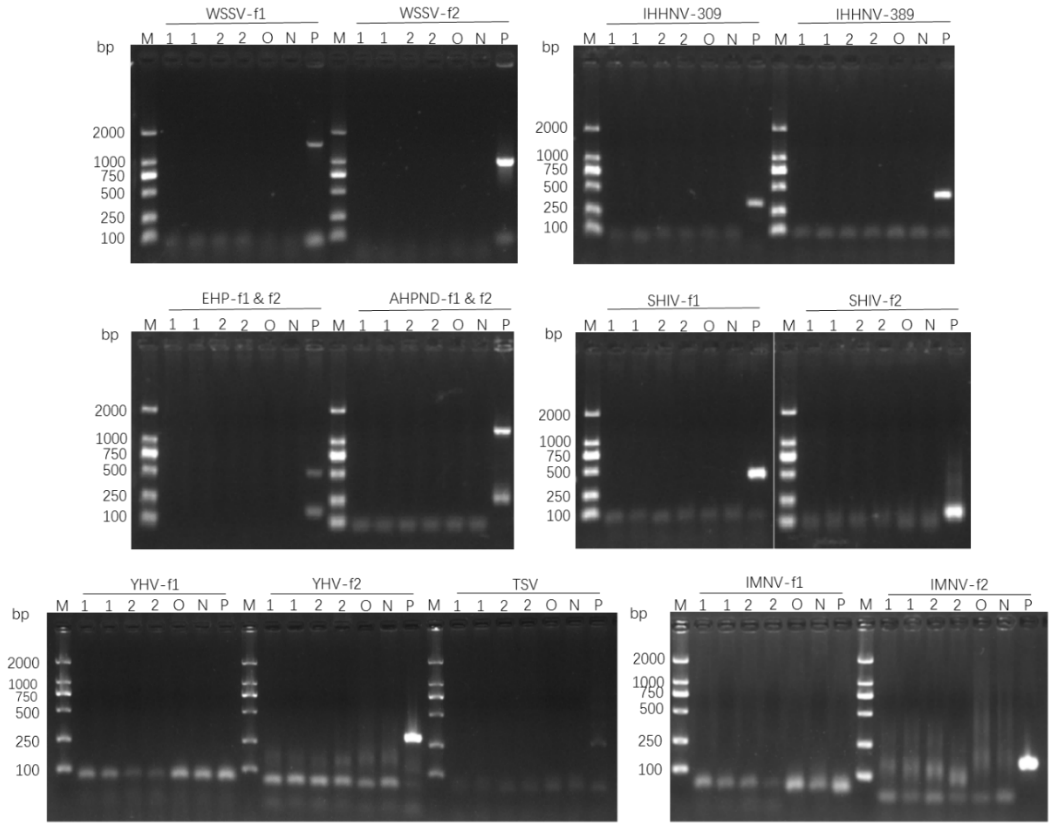

Figure S1. Electrophoretogram of molecular detection of eight common pathogens in the diseased individuals of the translucent post-larvae disease (TPD) case. M: molecular marker; P: positive control; N: negative control; S: sample. (f1) showed the first step PCR amplicon of nested PCR for each pathogen. (f2) showed the second step PCR amplicon of the nested PCR for each pathogen. AP4 primer set was used in the AHPND test. M: Molecular markers.

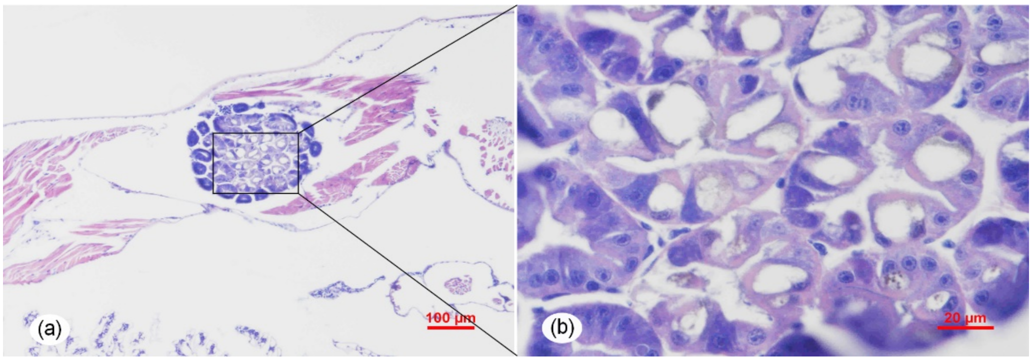

Figure S2. Histological sections of the normal shrimp from rearing tanks affected by TPD. (a) micrograph of low magnification. (b) The magnified micrographs of the area in the black frames in (a). Note the intact hepatopancreatic tubules (HTs) and epithelial cells of HTs. Scale bars = (a) 100 μm, (b) 20 μm.

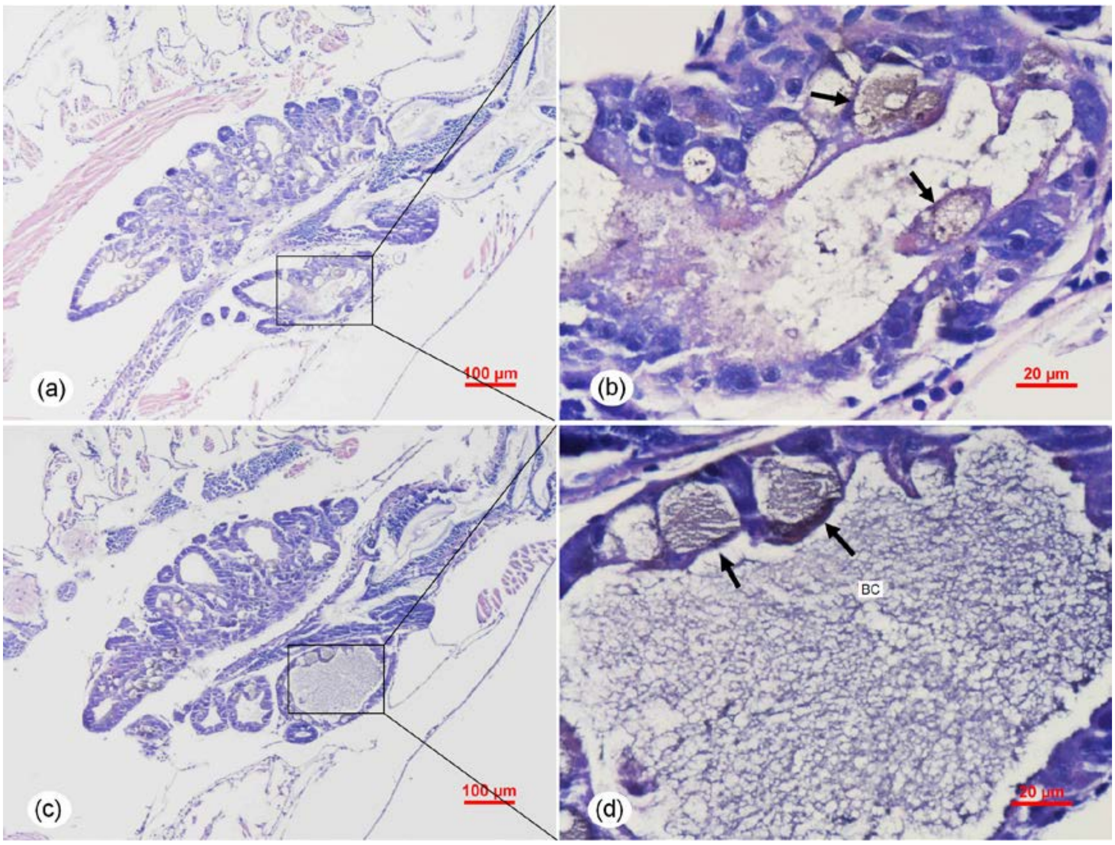

Figure S3. Histological sections of the hepatopancreatic (HP) tubules from the shrimp natural infected with TPD. (a, b) The hepatopancreas tubules infected by bacteria showed melanization (black arrows). (c, d) The massive bacterial colonization (BC) in epithelial cells and tubule lumens of the HP. Scale bars = (a) 100 μm, (b) 20 μm, (c) 100 μm, (d) 20 μm.

Supplementary Table 1. The results of biochemical test for *Vp*-JS20200428004-2 by API 20NE.

Parameters Tested	24 h	48 h
NO_3	+/+	+/+
TRP	+	+
GLU	+	+
ADH	-	-
URE	-	-
ESC	+	+
GEL	+	+
PNPG	-	-
GLU	+	+
ARA	-	-
MNE	+	+
MAN	-	+
NAG	+	+
MAL	-	+
GNT	+	+
CAP	-	-
ADI	-	-
MLT	+	+
CIT	-	-
PAC	-	-
OX	+	+
